# IL-17A Drives Oxidative Stress and Cell Growth in A549 Lung Epithelial Cells: Potential Protective Action of Oleuropein

**DOI:** 10.3390/nu16132123

**Published:** 2024-07-03

**Authors:** Angela Marina Montalbano, Caterina Di Sano, Giusy Daniela Albano, Mark Gjomarkaj, Fabio Luigi Massimo Ricciardolo, Mirella Profita

**Affiliations:** 1 Institute of Translational Pharmacology—National Research Council of Italy (IFT-CNR), 90146 Palermo, Italy; angelamarina.montalbano@cnr.it (A.M.M.); caterina.disano@cnr.it (C.D.S.); mark.gjomarkaj@cnr.it (M.G.); fabioluigimassimo.ricciardolo@unito.it (F.L.M.R.); 2 Department of Clinical and Biological Sciences, University of Turin, 10043 Turin, Italy

**Keywords:** IL-17A, lung diseases, oxidative stress, epithelial cells, Oleuropein

## Abstract

IL-17A drives inflammation and oxidative stress, affecting the progression of chronic lung diseases (asthma, chronic obstructive pulmonary disease (COPD), lung cancer, and cystic fibrosis). Oleuropein (OLP) is a polyphenolic compound present in olive oil and widely included in the Mediterranean diet. It exerts antioxidant and anti-inflammatory activities, oxidative stress resistance, and anticarcinogenic effects with a conceivable positive impact on human health. We hypothesized that OLP positively affects the mechanisms of oxidative stress, apoptosis, DNA damage, cell viability during proliferation, and cell growth in alveolar epithelial cells and tested its effect in a human alveolar epithelial cell line (A549) in the presence of IL-17A. Our results show that OLP decreases the levels of oxidative stress (Reactive Oxygen Species, Mitochondrial membrane potential) and DNA damage (H2AX phosphorylation-ser139, Olive Tail Moment data) and increases cell apoptosis in A549 cells exposed to IL-17A. Furthermore, OLP decreases the number of viable cells during proliferation, the migratory potential (Scratch test), and the single cell capacity to grow within colonies as a cancer phenotype in A549 cells exposed to IL-17A. In conclusion, we suggest that OLP might be useful to protect lung epithelial cells from oxidative stress, DNA damage, cell growth, and cell apoptosis. This effect might be exerted in lung diseases by the downregulation of IL-17A activities. Our results suggest a positive effect of the components of olive oil on human lung health.

## 1. Introduction

Th17 cells are a subtype of CD4^+^ T-cells with pro-inflammatory activities. Naive CD4^+^ T-cells differentiate into Th17 cells under the effects of a variety of cytokines (IL-6, IL-1β, IL-21, IL-23, and TGFβ) involved in airway inflammation. Th17 cells are present in the epithelium of patients with severe chronic inflammatory airway diseases. They drive the outcome of pulmonary infections, allergies, and pro inflammatory injuries by secreting IL-17A [1]. Th17 cells exert a protective activity toward the host in healthy subjects, although excessive production of IL-17A during chronic inflammation may promote the development of autoimmune diseases and cancer. In vitro and ex vivo studies show a specific role of IL-17A in tissue renewal and tissue damage of epithelial cells in the proximal and distal airways of patients with COPD [2]. Furthermore, its powerful proinflammatory and prooxidative actions stimulate chronic inflammatory diseases to proceed toward cancer, promoting tumor growth and metastasis [3,4,5,6]. Recent studies show that IL-17A overexpression has tumorigenic activities in non-small cell lung cancer (NSCLC) in mice [7,8,9] and it is associated with genomic instability in bronchial epithelial cells from the lung tissue of smokers and non-smokers [3]. IL-17A contributes to the DNA damage response (DDR) and chromosome breakage forming γH2AX foci in human bronchial epithelial [10] and pancreatic cells [11]. These data suggest the implication of IL-17A in cancer development [12]. 

The diet intake of antioxidants reduces the risk of the development of a variety of tumors (colorectal, lung, breast, stomach, and prostate) [13]. The habits of the populations living in Mediterranean countries include the consumption of foods with elevated contents of total fat, such as vegetables, fruits, nuts, fish, and olive oil. These populations show a lower rate of mortality related to colon cancer in comparison to Northern European or other Western countries’ populations [14,15]. Olive oil derives from olive tree fruits named Olea europaea. It is composed of fatty acids, vitamins, and polyphenols [16]. Oleuropein (OLP), vanillic acid, gallic acid, phenolic alcohols (tyrosol, hydroxytyrosol), secoiridoids (Oleocanthal), lignans (pinoresinol), and flavones (luteolin) are phenolic acid derivatives present in the olive oil. OLP and other bisphenols have attracted the interest of the food industry. Their antimicrobial and antioxidant properties are useful to formulate new functional products able to increase nutritional intake (dietary intake) or shelf life [17,18]. Nutrigenomic studies indicate that OLP exerts different positive effects by interfering with protein function and gene expression or by modifying cellular pathways relevant to the non-communicable diseases (NCDs) or, more in general, by positively affecting different pathological processes at molecular levels [19].

Lung cancer has a high incidence and mortality rate in the world, and non-small cell lung cancer (NSCLC) covers 80–85% of lung cancer cases [20]. Patients with NSCLC are poorly responsive to conventional therapeutic strategies including surgery, chemotherapy, and radiotherapy, with a less than 15% 5 years survival [21]. A strong relationship between inflammation and tumor development has been recently identified and both are positively correlated with tumor metastasis, neovascularization, and tumor immunity [22]. High-doses of OLP have a relevant impact on cisplatin-induced oxidative stress, genotoxicity and pathological changes in rat stomach and lung [23]. OLP downregulates tissue infiltration of macrophages and neutrophils by blocking OVA inhalation and ICAM-1, F4/80, CD68, and CD11b expression induced by cigarette smoke in mouse lungs [24]. However, the anticancer activities, the mechanisms of action, and the therapeutic effects of OLP remain unknown in human lung. Many animal studies encourage to study these mechanisms in human models.

The present study hypothesized that OLP positively interferes with the cellular and molecular mechanisms generated by high concentrations of IL-17A in the lung. We have investigated the activity of IL-17A in an in vitro model of alveolar basal epithelial cells (A549 cell line) cultured in the presence or absence of OLP in order to mimic the beneficial effect of a healthy diet including olive oil in lung diseases. We studied the mechanisms of: (1) oxidative stress (Reactive Oxygen Species (ROS) and mitochondrial membrane potential (JC1) production; (2) cell death including cell-apoptosis; (3) DNA damage (H2AX phosphorylation-ser139 and cell comet formation due to DNA fragmentation); (4) cell viability during proliferation, and (5) migratory potential of the cells (by Scratch test), and the single cell capacity to proliferate within a colony (like a cancer phenotype).

## 2. Materials and Methods

### 2.1. Chemicals and Biochemicals

Dulbecco’s Modified Eagle’s Medium (DMEM) High Glucose with Sodium Pyruvate, without L-Glutamine (cat. n. ECB7501L), Fetal Bovine Serum (FBS) EU Approved and heat-inactivated (cat. n. ECS0180L), L-Glutamine (cat. n. ECB3000D), non-essential amino acids 100X (cat. n. ECB3054D), Gentamicin Sulfate 50 mg (cat. n. 17518z), and Trypsin-EDTA (cat. n. ECB3052D), were purchased from Euroclone (Milan, Italy). Recombinant Human IL-17/IL-17A (cat. n. 7955) was purchased from R&D System a Brand Bio-Techne (Minneapolis, MN, USA), Oleuropein (cat. n. 12247) and Dimethyl Sulfoxide (DMSO) (cat. n. D2650) were purchased from Sigma–Aldrich Inc., (Milan, Italy). Mito Probe JC-1 Assay kit (cat. n. M34152) (5,5′,6,6′-tetrachloro-1,1′,3,3′-tetraethyl-benzimidazolo-carbocyanineiodide) and 2′,7′-dichloro-difluorescein diacetate (DCF-DA) were purchased from Molecular Probes, Inc., (Eugene, OR, USA). Cell Titre Aqueous Solution Reagent Kit was purchased from Promega (Madison, WI, USA). Annexin V FITC apoptosis detection Kit (cat. n. BMS500FI/300) was purchased from Bender Med Systems GmbH (Wien, Austria). 

### 2.2. Cell Cultures and Stimuli

Human alveolar adenocarcinoma cell line A549 (alveolar basal epithelial cell line) CCL-185 was purchased from the American Type Culture Collection (ATCC) (Rockville, MD, USA). A549 cells were plated (2 × 10^5^ cells/well) in standard six-well plates (submerged condition) in a suitable medium at 10% FBS and grown to confluence (70–80%) as previously described [2].

IL-17A was dissolved in PBS to obtain stock solutions of 100 µg/mL. OLP was dissolved in DMSO to obtain stock solutions of 200 mM. The IL-17A and OLP final concentrations were subsequently diluted in the growth medium. The effects of IL-17A or OLP were tested using a range of concentrations as previously described [8,25,26]. OLP was added to the cells 1 h before IL-17A stimulation. DMSO concentrations did not exceed 0.1% in cell cultures. 

### 2.3. Detection of Intracellular Reactive Oxygen Species (ROS) and Mitochondrial Membrane Potential (JC1) Measurement

IL-17A was added to the cell cultures at various concentrations (10, 20, and 50 ng/mL) for 4 h. After that, considering the results of the dose response of ROS and JC-1 production, the cells were stimulated with IL-17A 50 ng/mL in the presence or absence of OLP 50 μM for 4 h. Subsequently, A549 cells were trypsinized, washed in PBS, and incubated with DCF-DA 1 µM in PBS for 10 min (dark room, 25 °C). DCF-DA enters the cells, and the activity of ROS hydrolyzes in DCF. The cell death was converted into a nonfluorescent molecule following deacetylation mediated by esterases, which was subsequently oxidized by ROS into the fluorescent compound DCF. After washing, the fluorescent DCF cells were resuspended in PBS and analyzed by flow cytometry using a FACSCalibur™ flow cytometer (Becton Dickinson, Mountain View, CA, USA) as previously described [27,28]. Gating on the cells was performed excluding debris and using forward and sideways scatter patterns. The mitochondrial function was assessed in the same cell samples by membrane potential analysis using MitoProbe JC-1 Assay kit (Molecular Probes, Inc., Eugene, OR, USA), a lipophilic cationic dye, as previously described [2]. JC-1 locates itself into the mitochondrial inner membrane forming either aggregates (reflecting a high membrane potential as indicated by red fluorescence) or monomers (reflecting a low membrane potential and fluorescing green), depending on membrane potential. The fluorescence of 10,000 cells was recorded upon excitation at a fluorescent emission shift of JC-1 from Red (~590 nm) to green (~529 nm) and evaluated by FACSCalibur™ flow cytometer with 488 excitations lasers as previously described [27].

### 2.4. Cell Apoptosis

The culture medium was replaced when the cells reached confluence, and A549 cells were stimulated for 72 h with IL-17A at various concentrations (50 and 100 ng/mL). In this case, we extended the stimulation of the cells up to IL-17A at 100 ng/mL to verify that 50 ng/mL was the maximal possible effect on cell apoptosis. Subsequently, the cells were stimulated with IL-17A 50 ng/mL in the presence or absence of OLP 50 μM for 72 h. At the end of stimulation, the cells were stained with a solution containing a mixture of Annexin V FITC in binding buffer 1×. Cell apoptosis was assessed as previously described [2,27].

### 2.5. γH2AX by Flow Cytometry

Oxidative stress affects H2AX phosphorylation (γH2AX). According to our results on ROS/JC-1 and cell apoptosis, we selected the dose of IL-17A 50 ng/mL to study the levels of γH2AX expression and γH2AX foci formation within the nucleus. After the confluence of the cells, the medium was replaced, and A549 cells were incubated with IL-17A 50 ng/mL for 72 h in the presence or absence of OLP 50 μM. The cells were then starved and fixed in paraformaldehyde 4% (20 min RT), permeabilized with Permeabilization buffer for 20 min at RT, washed in PBS, and incubated overnight at 4 °C with primary rabbit polyclonal antibody anti-phosphor-H2AX (Merk Millipore, Temecula, CA, USA) (cat. n. 07-164) to identify the presence of OLP-induced and/or IL-17A-induced DNA double strand breaks. At the end of incubation, samples were rinsed and incubated with the secondary antibody Anti Rabbit FITC (Sigma-Aldrich, Inc., Milan, Italy) (cat. n. F7512) for 1 h at RT. Cells were then rinsed and resuspended in PBS and analyzed by a FACSCalibur™ flow cytometer (Becton Dickinson, Mountain View, CA, USA). Fluorescence-positive cells were quantified. Percentages of positive cells for γH2AX were determined from forward (FS) and sideways (SS) scatter patterns, after gating on the cells, excluding debris. Non-specific binding and background fluorescence were quantified by analyzing negative control.

### 2.6. Immunofluorescence of γH2AX Foci Formation

The detection of γH2AX foci was used to identify the DNA double-strand breaks as previously described [16]. A549 cells were cultured as described in the previous paragraph and then fixed in 4% paraformaldehyde for 10 min, permeabilized with 0.2% Triton X-100 for 5 min and blocked with 1% BSA for 1 h. A549 cells were incubated with a rabbit polyclonal anti–phospho-H2AX (1:100 dilution) overnight at 4 °C and then incubated with anti-rabbit FITC for 1 h. Finally, the cells were treated with Hoechst Sigma-Aldrich, Inc., Milan, Italy) (cat-n. 14533) (diluted 1:1000 in PBS for 10 min at room temperature) followed by wash in PBS to perform the nuclei stain. The slides were mounted using Vectashield mounting medium (Vector Laboratories, Burlingame, CA, USA) (cat. n. H-1000), and the fluorescence was visualized using a laser-scanning microscope ZEISS (Oberkochen, Germany) at a final magnification of 63×, Scale bar 20 µm. 

### 2.7. DNA Damage Analysis

γH2AX is related to the mechanism of DNA damage. According to our results on γH2AX, we incubated A549 cells with IL-17A 50 ng/mL for 72 h in the presence or absence of OLP 50 μM to study DNA damage by using Comet Assay as previously described [27]. The cells were then layered on one-third frosted slides pre-coated, OxiSelect™ Comet Assay Kit (Cell Biolabs, Inc., San Diego, CA, USA) (cat. n. STA-35) and subsequently lysed and subjected to electrophoresis at 0.7 V/cm for 30 min. Comet images were acquired at 10× magnification by using a fluorescence microscope ZEISS (Oberkochen, Germany) and subjected to image analysis using Image J software (v1.53e). The DNA damage was quantified as previously described [27]. 

### 2.8. MTS AssayA549 Cells 

Cells were cultured at a density of 1 × 10^3^ cells per wells in a 96-well plate in 200 μL DMEM 10% FBS and then incubated with IL-17A (50 and 100 ng/mL). We extended the incubation of the cells to an IL-17A concentration of 100 ng/mL to verify that 50 ng/mL is the maximal possible effect on the number of viable cells during proliferation. Furthermore, A549 cells were incubated with IL-17A 50 ng/mL in the presence or absence of OLP 50 μM for 72 h. Cell viability was determined in vitro by 3-(4,5-dimethylthiazol-2-yl)-5-(3-carboxymethoxyphenyl)-2-(4-sulfophenyl)-2H-tetrazolium MTS assay, using the CellTiter 96^®^ Aqueous One Solution Cell Proliferation Assay kit (Promega, Madison, WI, USA), according to the manufacturer’s instruction, as previously described [27]. A549 is a cell line growing by proliferation in monolayers. MTS assay is a colorimetric method used for determining the number of viable cells during proliferation or cytotoxicity.

### 2.9. Clonogenic Assay 

In agreement with data obtained with the MTS assay, a viable A549 cell line (2 × 10^5^) was seeded into 6-well plate with DMEM 10% FBS and cultured with IL-17A 50 ng/mL for 72 h in the presence or absence of OLP 50 µM. Subsequently, cells were harvested and seeded (5 × 10^4^) on the upper layer of agar in 35 mm Petri dishes (Falcon Becton Dickinson, Franklin Lakes, NJ, USA) to perform clonogenic assay. Briefly, the lower layer of agar was prepared in 35 mm Petri dishes using a medium supplemented with heat-inactivated (56 °C, 30 min) FBS in 0.5% agarose, and the upper layer was prepared with 0.3% agarose. Finally, the cells were incubated for 21 days at 37 °C in an atmosphere containing 5% CO_2_. Fresh medium DMEM 10% FBS (500 μL) was weekly added to the upper layer of agar to prevent desiccation. At the end of incubation, the growth of the colonies was counted under an inverted phase-contrast microscope (Leitz, Wetzlar, Germany). Colonies were defined as cell aggregates composed of at least 40 cells. Data were generated from four biological replicates of three experiments. The results are expressed as Number of Colonies ± SD [29].

### 2.10. Long Term Exposure of A549 Cells

The confluent cells were long-term exposed to IL-17A 50 ng/mL in the presence or absence of OLP 50 μM. Briefly, during the first 7 days, the cells were cultured with and without OLP, and subsequently exposed (or not exposed) to IL-17A 50 ng/mL for additional 7 days. At the end of this 14 days incubation, cells were detached, re-plated, and fresh stimuli were again added every 72 h for additional 14 days in order to test the effects on A549 cells chronically exposed to OLP and IL-17A. The viability of the cells exposed to stimuli was analyzed by blue dye exclusion assay. 

### 2.11. Cell Migration Assay (Scratch/Wound Healing Assay)

A549 cells, long-term (14 days) exposed to the stimuli were seeded on 6-well plates at a density of 1 × 10^6^ until confluence. Artificial wounds were created in the confluent cell monolayers using a sterile 200 μL pipette tip, and the non-adherent cells were washed off with PBS. Cells were then treated with IL-17A (50 ng/mL), alone or in combination with OLP 50 μM, for 72 h. The wounds (scratch lines) were then observed and photographed, at time 0 and 72 h after treatment, using a light microscope at 10× objective magnification [30,31]. The wound area was measured using Image J software. The percentage of gap area reduction was obtained by the measurement of wound area at 72 h (T_72_) compared with the wound area at T_0_ (set to 100% Wound Area). 

### 2.12. Statistical Analysis

All data were generated from independent biological replicates and expressed as means ± standard deviation (SD). Results from Cytofluorimetric analysis and MTS assay were expressed as fold change obtained choosing baseline cells as reference sample (assumed to be equal to 1) to uniform the data of all experiments. Analysis of variance (ANOVA) for multiple comparisons was corrected with post hoc Fisher’s test. ANOVA test showed that there is a statistically significant difference between groups of data, and that there are statistically significant differences between the means of each group of experimental conditions. as defined with post hoc Fisher’s test. All statistical analyses were performed using Stat View^®^ 5 software (SAS institute Inc., Cary, NC, USA). A *p* value < 0.05 was statistically significant. 

## 3. Results 

### 3.1. IL-17A Drives ROS Production and Mitochondrial Injury in A549 Cells: Effect of OLP

**Figure 1:**
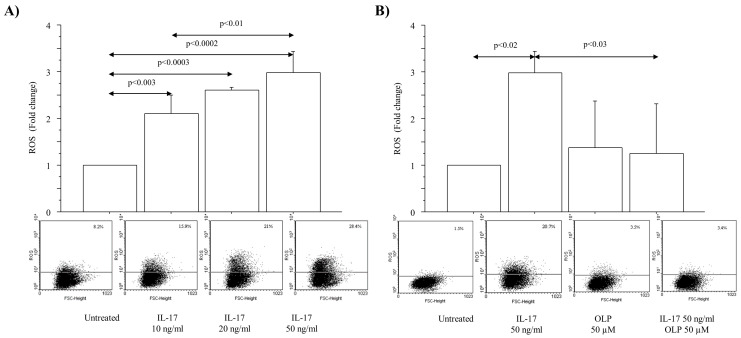
Effect of IL-17A on ROS production in A549 cells. ROS production in A549 cells (**A**) treated with different concentrations of IL-17A (10, 20, 50 ng/mL) for 4 h; (**B**) treated with IL-17A 50 ng/mL for 4 h in the presence or absence of OLP 50 μM added to the cells 1 h before. Representative flowcytometric analyses are shown. Bars represent mean ± S.D of Gated (%) expressed as fold change (n = 3). Statistical analysis was performed by ANOVA test with Fisher’s correction for multiple comparisons. Significance is accepted at *p* < 0.05. Abbreviations: ROS, Reactive Oxygen Species; IL-17A, Interleukin-17A; OLP, Oleuropein, ng, nanograms; µM, micromolar.

ROS production (expressed as fold change) was significantly increased in A549 cells cultured with a range of IL-17A concentrations (10, 20 and 50 ng/mL) for 4 h (*p* < 0.003, *p* < 0.0003, *p* < 0.0002, respectively) (Figure 1A). In light of the dose–response experiments, IL-17A at a concentration of 50 ng/mL was selected to perform the experiments with OLP. OLP 50 μM significantly reduced the ROS production in untreated cells as well as in the cells cultured with IL-17A 50 ng/mL for 4 h in comparison to the cells treated with IL-17A 50 ng/mL alone (Figure 1B). 

**Figure 2:**
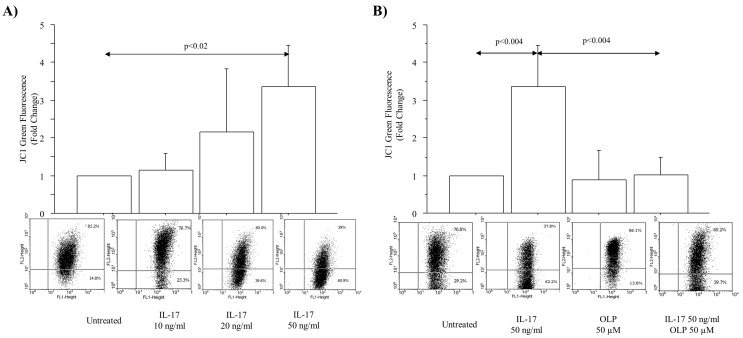
Effect of OLP on JC-1 production in IL-17A-treated A549 cells. JC-1 (monomers) green fluorescence (**A**) in cells cultured with different concentrations of IL-17A (10, 20, 50 ng/mL) for 4 h, and (**B**) in the cells treated with 50 ng/mL of IL-17A for 4 h in the presence or absence of OLP 50 μM added to the cells 1 h before. Representative flowcytometric analyses are shown. Bars represent mean ± S.D of Gated (%) expressed as fold change (n = 3). Statistical analysis was performed by ANOVA test with Fisher’s correction for multiple comparisons. Significance is accepted at *p* < 0.05. Abbreviations: JC-1, 5,5′,6,6′-tetrachloro-1,1′,3,3′-tetraethyl-benzimidazolo-carbocyanineiodide; IL-17A, Interleukin-17A; OLP, Oleuropein, ng, nanograms; µM, micromolar.

The levels of JC-1 (monomers) green fluorescence (expressed as change from baseline) were significantly increased in A549 cells treated with IL-17A 50 ng/mL (*p* < 0.02) (Figure 2A). Again, in light of the dose–response tests, IL-17A at a concentration of 50 ng/mL was selected to perform the experiments with OLP. OLP 50 μM significantly reduced the intracellular JC-1 monomer production in untreated cells and in the cells cultured with IL-17A 50 ng/mL for 4 h in comparison to the cells treated with IL-17A 50 ng/mL alone (Figure 2B). These data suggest the concept that OLP exerts an antioxidant action in airway epithelial cells exposed to IL-17A. 

### 3.2. IL-17A Drives Cell Apoptosis in A549 Cells: Effect of OLP

**Figure 3:**
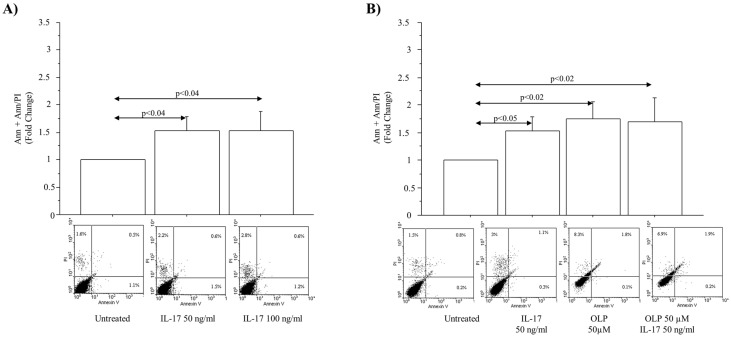
Effect of IL-17A and OLP on cell apoptosis in A549 cells. Apoptosis (Anx^+^ + Anx^+^PI^+^ cells) in the cells (**A**) A549 cells incubated with IL-17A 50 and 100 ng/mL for 72 h; (**B**) A549 cells incubated with IL-17A (50 ng/mL) for 72 h in the presence or absence of OLP (50 μM) added to the cells 1 h before. Representative flowcytometric analyses. Bars represent mean ± S.D of Gated (%) of apoptotic cells (Anx^+^ + Anx^+^PI^+^) expressed as fold change (n = 3). Statistical analysis was performed by ANOVA test with Fisher’s correction for multiple comparisons. Significance is accepted at *p* < 0.05. Abbreviations IL-17A, Interleukyne-17A; OLP, Oleuropein; ng, nanograms; µM, micromolar.

The levels of cell apoptosis (Anx^+^PI^−^ + Anx^+^PI^+^) (early + late apoptosis) (expressed as fold change) were significantly increased in A549 cells cultured with different concentrations of IL-17A (50 and 100 ng/mL) (*p* < 0.04, and *p* < 0.04, respectively) (Figure 3A). IL-17A 50/ng/mL was again selected to perform the experiments with OLP 50 µM. OLP 50 µM significantly increased the percentage of apoptotic cells (Anx^+^PI^−^ + Anx^+^PI^+^) (early + later apoptosis) in untreated cells (*p* < 0.02) and in the cells cultured with IL-17A (50 ng/mL) in comparison to cells treated with IL-17A (50 ng/mL) alone (*p* < 0.05) (Figure 3B).

### 3.3. IL-17A Drives DNA Damage/Repair Mechanisms in A549 Cells: Effect of OLP

**Figure 4:**
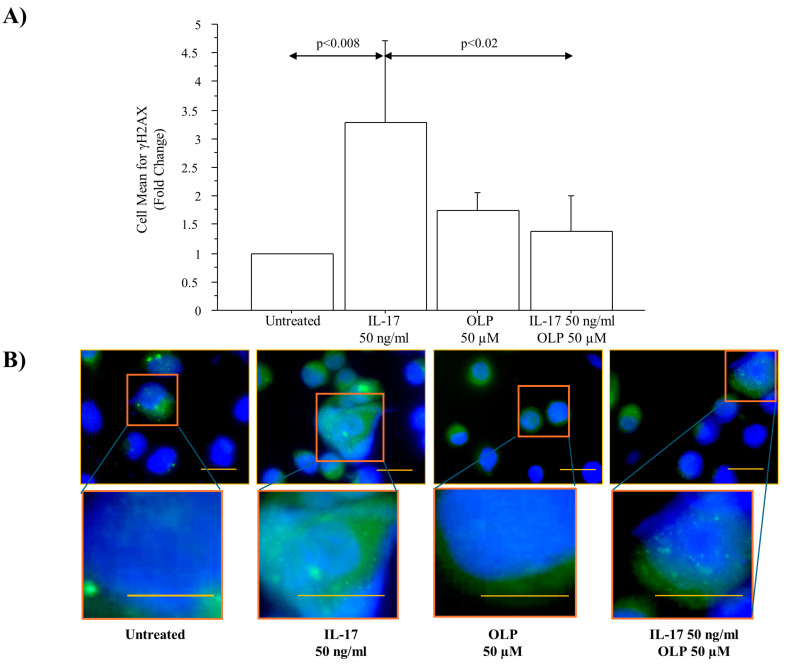
Effects of IL-17A and OLP (50 µM) on γH2AX activities in A549 cells. (**A**) The cells were incubated with IL-17A (50 ng/mL) for 72 h in the presence or absence of OLP 50 µM added to the cells 1 h before. Flow Cytometry analysis of γH2AX phosphorylation (ser139). Bars represent mean ± S.D of cell mean of fold change (n = 3). Statistical analysis was performed by ANOVA test with Fisher’s correction for multiple comparisons. Significance is accepted at *p* < 0.05. (**B**) Immunofluorescence analysis of γH2AX foci. A representative image of γH2AX immunofluorescence for each experimental condition was acquired and shown at 63× magnification. Scale bar 20 µm. Cell nuclei were stained with Hoechst 33342 (Blue fluorescence). Abbreviations: IL-17A, Interleukin-17A; OLP, Oleuropein; γH2AX, Histone H2AX phosphor-ser139; ng, nanograms; µM, micromolar.

**Figure 5:**
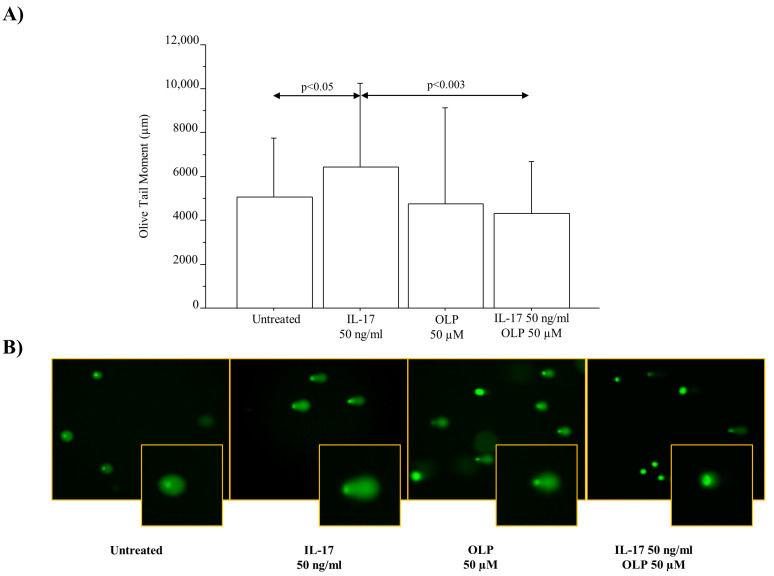
Genomic DNA Damage detected by the alkaline Comet Assay in A549 cells stimulated with IL-17A 50 ng/mL for 72 h in the presence or absence of OLP 50 µM added to the cells 1 h before. (**A**) Olive Tail Moment measurement. In total, 50 randomly selected cells were analyzed per sample. Bars represent mean ± S.D of Olive Tail Moment measurement. Statistical analysis was performed by ANOVA test with Fisher’s correction for multiple comparisons. Significance is accepted at *p* < 0.05. (**B**) A representative image of comet assay for each experimental condition was shown to indicate DNA damage. Abbreviations: IL-17A, Interleukin-17A; OLP, Oleuropein; ng, nanograms; µM, micromolar.

The levels of γH2AX phosphorylation expressed as fold change were significantly increased in A549 cells cultured with IL-17A 50 ng/mL for 72 h (*p* < 0.008). OLP 50 µM significantly decreased the γH2AX phosphorylation in the cells incubated with IL-17A 50 ng/mL in comparison to the cells incubated with IL-17A alone (*p* < 0.02) (Figure 4A). In agreement with these findings and the protective effects of OLP, a relevant number of γH2AX foci was detected in the cells incubated with IL-17A 50 ng/mL for 72 h rather than in untreated cells or in cells exposed to IL-17A and pre-treated with OLP 50 µM (Figure 4B). Furthermore, we analyzed the DNA damage by Comet Assay to identify the broken DNA. Interestingly, IL-17A 50 ng/mL significantly increased the Olive Tail Moment in the A549 cells in comparison to untreated cells (*p* < 0.05). Finally, the pre-treatment of the cells with OLP 50 µM significantly reduced the Olive Tail Moment in the cells treated with IL-17A 50 ng/mL when compared to the cells treated with IL17A alone (*p* < 0.003) (Figure 5).

### 3.4. IL-17A Drives Cell Viability during Proliferation: Effect of OLP

**Figure 6:**
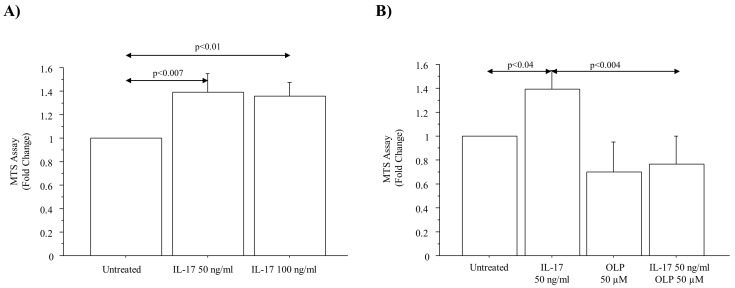
Effects of IL-17A and OLP on cell viability in A549 cells. Viability by MTS assay in A549 cells incubated with (**A**) IL-17A 50 and 100 ng/mL for 72 h; (**B**) IL-17A (50 ng/mL) for 72 h in the presence or absence of OLP 50 µM added to the cells 1 h before. Bars represent mean ± S.D. expressed as fold change (n = 3). Statistical analysis was performed by ANOVA test with Fisher’s correction for multiple comparisons. Significance was set at *p* < 0.05. Abbreviations: IL-17A, Interleukyne-17A; OLP, Oleuropein; ng, nanograms; µM, micromolar.

The number of viable cells during proliferation measured by the MTS assay (expressed as fold change) was significantly increased in A549 cells incubated with different concentrations of IL-17A (50 and 100 ng/mL) (*p* < 0.007; *p* < 0.01, respectively) for 72 h (Figure 6A). IL-17A 50 ng/mL was selected to perform the experiments with OLP. OLP 50 µM showed a significant ability to reduce the number of viable cells during proliferation in untreated cells or in the cells treated with IL-17A 50 ng/mL for 72 h in comparison to the cells treated with IL-17A (50 ng/mL) alone (*p* < 0.04 and *p* < 0.004, respectively) (Figure 6B). 

### 3.5. IL-17A Drives Colony Formation and Invasiveness in A549 Cells and OLP Downregulates These Phenomena

**Figure 7:**
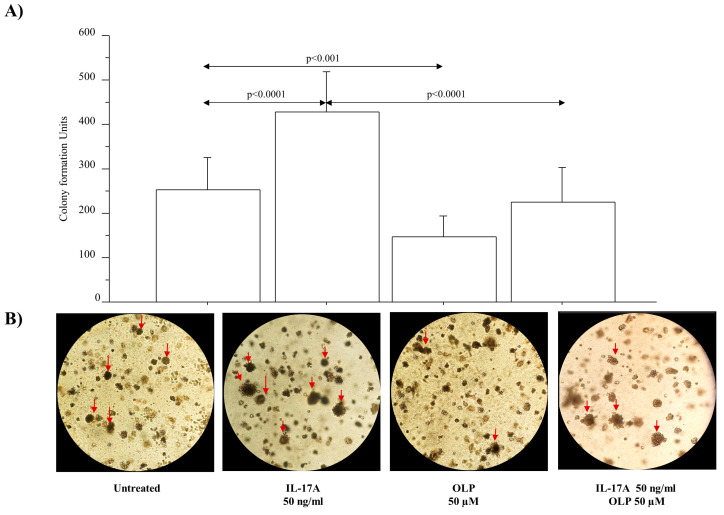
Effects of IL-17A and/or OLP, alone or in combination, on colony growth in A549 cells. (**A**) Cells treated with IL-17A 50 ng/mL in the presence or absence of OLP 50 µM, for 72 h and used to evaluate the long-term colony growth (21 days) by clonogenic assay. Data are expressed as mean ± SD of number of colonies compared with untreated A549 cells (n = 3). Statistical analysis was performed by ANOVA test with Fisher’s correction for multiple comparisons. Significance was set at *p* < 0.05. (**B**) A representative image of colony formation assay for each experimental condition was acquired and shown. The arrows enclosed in the images indicate the colonies. Abbreviations: IL-17A, Interleukyne-17A; OLP, Oleuropein; ng, nanograms; µM, micromolar.

**Figure 8:**
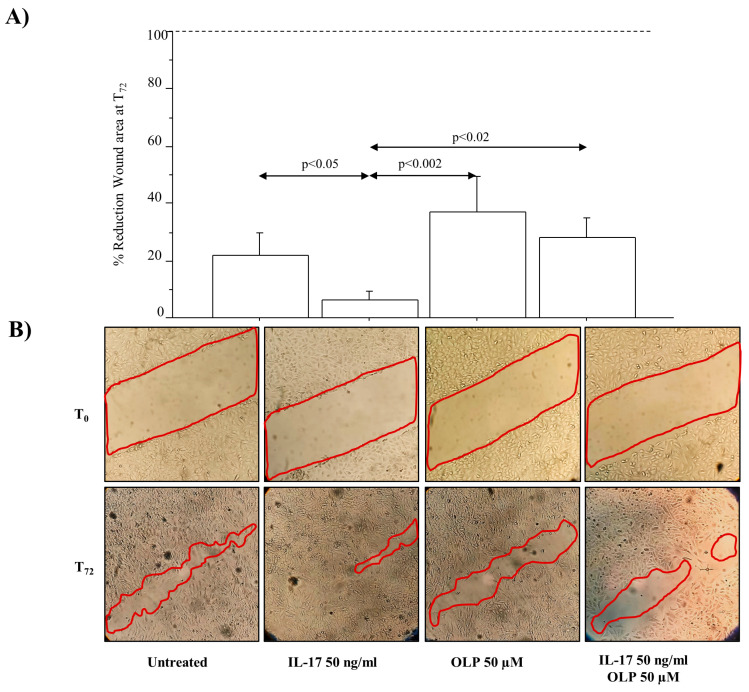
Effect of IL-17A, alone or in combination with OLP, on scratch test assay. A549 cells treated with IL-17A 50 ng/mL, alone or in combination with 50 μM OLP for 14 days (chronic exposure) and replated at the density of 1 × 10^6^ until confluence. A549 cells were then treated again with the stimuli and time 0 (T_0_) and after 72 h and the Wound healing (scratch) migration assay was performed and evaluated (T_72_). (**A**) Quantitative analyses of the migration assay are expressed as percentages of reduction in wound area after 72 h and compared to wound area at T_0_ (set to 100%). In the figure, the dash/red line defines the wound area. The wound was filled with migratory cells from both sides of the scratched area. Data are expressed as mean ± SD of area compared to untreated cells (n = 3). Statistical analysis was performed by ANOVA test with Fisher’s correction for multiple comparisons. Significance was set at *p* < 0.05. (**B**) A representative image of microscopy visualization for each experimental condition is shown at 10× magnification. Abbreviations: IL-17A, Interleukyne-17A; OLP, Oleuropein; ng, nanograms; µM, micromolar.

The colony growth was significantly increased in A549 cells treated with IL-17A 50 ng/mL in comparison to untreated cells (*p* < 0.0001). The colony growth was significantly reduced in A549 cells treated with OLP alone in comparison to untreated cells (*p* < 0.001). Furthermore, OLP significantly reduced the colony growth in A549 cells treated with IL-17A in comparison to the cells stimulated with IL-17A alone (*p* < 0.0001, respectively) (Figure 7). In addition, we evaluated the migration of A549 cells in a model of chronic exposure (14 days) to IL-17A by using a wound healing (scratch) assay. The wound was filled with migratory cells from both sides of the scratched area. A549 cells treated with IL-17A 50 ng/mL were more confluent than the untreated cells (*p* < 0.05) (Figure 7). The pre-treatment of the cells with OLP 50 µM significantly reduced A549 cell migration in the healing area in both cells treated with IL-17A and cells treated with OLP and IL-17A (*p*< 0.002 and *p*< 0.02, respectively) (Figure 8).

## 4. Discussion

We investigated the action of OLP on IL-17A mediated prooxidative, proinflammatory and pro-cancerogenic activities in the lung. OLP is a micronutrient belonging to the polyphenol family that is present in olive oil [3]. Our results demonstrate that IL-17A generates high levels of oxidative stress, apoptosis, and DNA damage (pH2AX expression and comet formation) in A549 cells (in vitro model) and that OLP has a potential positive action on the cellular dangerous proinflammatory and prooxidative activities of this cytokine involved in a variety of airway diseases. Although the weakness of this study is related to the fact that it has been performed using a cell line (A549), it creates the strength for future research perspectives aimed at studying the protective action of OLP in animal or lung organoid models or even in clinical settings.

Its slow but constant renewal makes the airway epithelium a dynamic tissue. After an injury, it spontaneously undergoes self-renewal. The airway epithelium is a physical barrier of the lung involved in fluid balance, modulation of metabolism, and clearance of inhaled agents and secretes many mediators within the lung. Most of these mediators affect the recruitment and activation of inflammatory cells, like Th17. Dysregulation of airway epithelial cell function, related to IL-17 production, might contribute to the pathogenesis of major lung diseases, including severe inflammatory diseases and cancer [32]. Preclinical studies show that IL-17A mediates cytokine and chemokine expression in structural epithelial cells, affecting neutrophilia, remodeling of the airway, loss of lung function and lung damage in COPD, cystic fibrosis, and asthma, as well as tumor proliferation and immunotherapy-resistance [33], cell invasion, metastasis, and angiogenesis [32]. Oxidizing agents are aggressive against a variety of biological components, including lipids, proteins, mitochondria, and DNA. In this manner, they promote metabolic disorders, inflammation, and cancer. Elevated levels of oxidative stress cause an increase in ROS and green fluorescent JC-1 monomers production due to a decrease in mitochondrial membrane potential (as a marker of mitochondrial dysfunction) [18]. It is well known that elevated levels of ROS and JC-1 generate damage in cell compartments, promoting cell apoptosis. Oxidative stress and inflammation lead to DNA damage, increasing the risk of cancer development [34]. One of the primordial cellular events involved in the DNA damage response (DDR), the index of a wide range of DNA lesions, is the phosphorylation of histone H2AX at serine 139 (termed γH2AX). Its expression in nuclei is used to specifically indicate DNA double-strand breaks [35]. γH2AX activity generates the formation of foci within the nucleus, which are involved in the mechanisms of DNA repair [36,37,38]. The assay of single-cell gel electrophoresis, known as “Comet Assay” is a technique useful to measure DNA strand breaks in individual mammalian cells [39]. In our study, we show that IL-17A affects the mechanisms of oxidative stress by increasing ROS and JC-1 production in A549 cells cultured in vitro in a monolayer model. Consequently, IL-17A increases cell-apoptosis, DNA damage, elevated levels of H2AX phosphorylation, foci, and comet formation in A549 cells. These data might indicate that IL-17A triggers oxidative stress and activates the cellular mechanisms of carcinogenesis by promoting DDR in lung epithelial cells. In agreement with this statement, we observed that IL-17A increases the number of cell viability during proliferation promoting, after a long-term exposure, the formation of cell colonies, and an increased rate of cell migration in A549 NSCLC cells. However, the mechanisms by which IL-17A generates the growth and development of NSCLC are still not well known [26]. These findings might be related to an increased proliferation rate, but further studies (i.e., cell cycle progression) are necessary to better clarify these phenomena.

A549 cells cultured in the presence of IL-17A show an increase in two, only apparently counteracting, cellular events: the number of viable cells during proliferation and cell apoptosis. In agreement with this, high concentrations of IL-17A (50 to 500 ng/mL) significantly increased cell proliferation in a dose-dependent manner by high-mobility group A1 (HMGA1) upregulation in A549 NSCLC cells [2,8]. Moreover, we observed that IL-17A promotes cell growth and migration, cellular events strongly associated with cell viability and proliferation. Recent published data describe that IL-17-mediated inflammation promotes cigarette smoke-induced genomic instability in mouse lungs [3]. According to these findings, we show here that IL-17A affects cell apoptosis together with cellular events associated with DNA damage and genomic instability, supporting the concept that IL-17 could play an important role in the development of lung cancer. We believe that these conflicting findings might be involved in the poor control of IL-17A related inflammatory events and their controversial role in cancer. Further studies might be necessary to better understand the IL-17A activities during inflammatory processes in the airway epithelial cells. Targeting IL-17-signaling might be considered a putative pharmacologic therapeutic interventions in chronic lung diseases and, eventually, in combination with additional immunotherapies, in lung cancer. However, up until today, clinical studies were not able to demonstrate the benefit of IL-17 cytokines as therapeutic targets in chronic lung diseases. Consequently, it is important to identify new pharmacological strategies useful to control IL-17A-mediated signal pathways [40].

Chemotherapies induce tumor cell death by the activation of cell apoptosis. Defining the mechanisms of action of drugs already used in clinical practice, and finding new pharmacological solutions is fundamental for the management of cancer [29,40,41,42]. OLP exerts pharmacological benefits as antioxidant, anti-inflammatory, antiatherogenic, anticancer, antimicrobial, and antiviral activities [43,44], and high-doses of OLP reduce cisplatin-induced oxidative stress, genotoxicity and pathological changes in rat stomach and lung [23]. It is a food supplement [45] involved in the inhibition of cell proliferation and in the induction of apoptosis in cancer cell lines via different mechanisms. OLP induces apoptosis, affecting a mitochondrial apoptotic cascade by the P38 MAP kinase pathway in A549 cells [25] or reduces ROS production by the PI3K/AKT pathway in Hepatocellular Carcinoma Cells [46]. Accordingly, we show here that OLP significantly reduces ROS and JC-1 in A549 cells stimulated with IL-17A and significantly increases the levels of apoptosis in A549 cells alone or pretreated with IL-17A. These findings support the concept that OLP present in olive oil (included in the Mediterranean diet) counteracts the mechanisms of oxidative stress generated by IL-17A in chronic airway diseases and, independently from its protective action on IL-17A-mediated effects, might improve the control of carcinogenesis by promoting lung epithelial cell death. These apparently conflicting effects might be generated by the activation of different, not yet known, mechanisms. Finally, we show that OLP reduces the levels of γH2AX (ser139), nuclear foci formation, and comet production in A549 cells treated with IL-17A. Taken together, these findings suggest that OLP positively regulates the mechanism of DNA damage/repair generated in the airway epithelial cells exposed to IL-17A. The different effects of OLP on the production of ROS/JC-1 and on the mechanisms of DNA damage/repair, compared with its effect on cell-apoptosis, might be explained by assuming that this natural compound regulates different cell activities by different signal pathways, dependently or independently from IL-17A. Further studies are necessary to better clarify the mechanisms by which OLP affects signal pathways and the related control of epithelial cell death during inflammatory processes in the lung. To confirm our flowcytometry data on cell apoptosis, it might be necessary to study the downstream targets (i.e., caspase 3) associated with the activities of OLP and IL-17A.

Cell viability is fundamental to evaluating the metabolic activities, survival, and death of the cells in response to various stimuli. OLP is known as an inhibitor of cell proliferation, invasion, migration, and as inductor of tumor cell death. It targets the oncogenic mechanisms in a variety of human malignancies [47,48]. Accordingly, we describe that OLP affects the metabolic activities of A549 cells stimulated with IL-17A, reducing the number of viable cells during proliferation. Furthermore, OLP counteracts the proliferative state, associated with the capability to form colonies (clonogenic assay), and invasiveness (scratch/wound healing assay) observed in A549 cells exposed to IL-17A for an extended period (chronic model). All these findings support the concept that OLP might inhibit epithelial cell transformation from a normal to a tumor cell phenotype. Based on these observations, it is conceivable that the habit of assuming olive oil contains polyphenolic compounds might be relevant as a promising beneficial and natural anticancer behavior.

The benefits of OLP (or other polyphenolic compounds) [49] and the involvement of IL-17A in the cellular and molecular mechanisms of inflammation and lung cancer development have been investigated in many in vitro studies at high concentrations (0 to 200 mM and 0–100 ng/mL, respectively) but never reached in vivo. The free forms of the phenolic compounds are not detected in plasma samples or are not exceeding 10 μM [50,51], while the serum levels of human IL-17A are in the order of a few picograms [52]. However, it is not unusual for immortalized cells to require higher doses of cytokines and molecules such as polyphenols to elicit the biological response observed in vivo. 

The detection of oxidation and anti-oxidation activities of polyphenol as OLP in biological systems is conditioned by metal or enzyme catalysis, membrane structure, molecule solubility and polarity, pH, water activity, bioavailability, and metabolism. All polyphenols interact with proteins/enzymes, as their hydroxyls of the benzoic group form hydrogen bonds with amino acids, interfering with their activities. However, the low levels of polyphenols and their metabolites in human blood and tissue might not support their potential and competitive antioxidant activity in vivo. To validate the use of polyphenols in the applications of preventive medicine, it is important to understand the effectiveness and chemistry of these natural compounds in the diet [53,54]. Therefore, further and addressed in vivo studies are needed to better validate the biological effects of the low dietary phenolic compounds at low plasma level concentrations and their relationship with IL-17A.

## 5. Conclusions

In conclusion, we describe the capability of IL-17A to induce oxidative stress, promoting the dysregulation of cell viability during proliferation, apoptosis, DNA damage response, and cell growth in an in vitro model of an A549 cell line (human alveolar adenocarcinoma). We suggest the protective action of OLP on IL-17A activity in epithelial cells. Indeed, our experimental approach defines OLP as a molecule with potential capabilities to reduce chromosomal damage and consequently to control various pathological aspects such as inflammation, tissue impairment, genetic anomalies, and carcinogenesis of the airways, leading to positive consequences for inflammatory and cancerogenic processes. Going into details, these data open future perspectives to study the specific mechanisms by which OLP exerts its protective actions and might underline the benefits and therapeutic implications of the Mediterranean diet for chronic lung diseases and cancer through the consumption of olive oil containing polyphenols. We used NSCLC cells (cell line A549), which represent 85% of lung cancer phenotypes. However, it might be useful to test the effects of OLP using further lung cancer cell lines to extend the knowledge to other cancer phenotypes. Moreover, it might be interesting to study the potential beneficial effect of OLP on normal alveolar epithelial cell growth and death (commonly missed in most studies like this) to test its safety/lack of damage in non-cancer conditions. Although the use of lung cancer cell lines could generate some difficulties in the interpretation of the results, it indeed opens up a future perspective of research to identify the potential protective activities of polyphenols.

This study was conducted in vitro, and the results should be interpreted with caution as they may not necessarily always mirror what happens in vivo. However, although epidemiological studies support the beneficial effect of OLP on human health, there is a need for a broader scope associated with an epidemiological approach, and this might help to better define the role of the Mediterranean diet, including OLP, in pulmonary diseases. From this point onward, there is a need for in vivo studies to further clarify the beneficial effects of polyphenols on human lung chronic diseases and lung cancer. 

## Data Availability

Data are contained within the figure article as box plot.

## Abbreviations

ATCC: American Type Culture Collection; CD4: Cluster of Differentiation 4; CFSE: carboxyfluorescein succinimidyl ester; COPD: chronic obstructive pulmonary disease; DCF: dichlorodihydrofluorescein; DCF-DA: 2′,7′-dichloro-difluorescein diacetate; DDR: DNA Damage Response; DMEM: Dulbecco’s Modified Eagle’s Medium High Glucose; DMSO: Dimethyl sulfoxide; DNA: Deoxyribonucleic acid; FBS: Fetal Bovine Serum; FITC: Fluorescein isothiocyanate; γH2AX: Histone H2AX phospho ser139; hrs: hours; ICAM-1: Intercellular Adhesion Molecule-1; IL-17A: Interleukin 17°; JC-1: (5,5′,6,6′-tetrachloro-1,1′,3,3′-tetraethyl-benzimidazolo-carbocyanineiodide); MEM: non-essential aminoacids; MEM: Eagle’s minimum essential medium; MMP: mitochondrial membrane potential; µM: micromolar; NCDs: non-communicable diseases; ng: nanograms; NK: Natural killer cells; NSCLC: non-small cell lung cancer; OLP: Oleuropein; PBS: Phosphate Buffered Saline; TGFβ: Tumor growth factor β; Th-17: T helper 17.

## References

[1] Song M., Liang J., Wang L., Li W., Jiang S., Xu S., Tang L., Du Q., Liu G., Meng H. (2023). IL-17A Functions and the Therapeutic Use of IL-17A and IL-17RA Targeted Antibodies for Cancer Treatment. Int. Immunopharmacol..

[2] Montalbano A.M., Riccobono L., Siena L., Chiappara G., Di Sano C., Anzalone G., Gagliardo R., Ricciardolo F.L.M., Sorbello V., Pipitone L. (2015). Cigarette Smoke Affects IL-17A, IL-17F and IL-17 Receptor Expression in the Lung Tissue: Ex Vivo and in Vitro Studies. Cytokine.

[3] Dharshini L.C.P., Rasmi R.R., Kathirvelan C., Kumar K.M., Saradhadevi K.M., Sakthivel K.M. (2023). Regulatory Components of Oxidative Stress and Inflammation and Their Complex Interplay in Carcinogenesis. Appl. Biochem. Biotechnol..

[4] Cao C., Tian B., Geng X., Zhou H., Xu Z., Lai T., Wu Y., Bao Z., Chen Z., Li W. (2021). IL-17-Mediated Inflammation Promotes Cigarette Smoke-Induced Genomic Instability. Cells.

[5] Xiang T., Long H., He L., Han X., Lin K., Liang Z., Zhuo W., Xie R., Zhu B. (2015). Interleukin-17 Produced by Tumor Microenvironment Promotes Self-Renewal of CD133+ Cancer Stem-like Cells in Ovarian Cancer. Oncogene.

[6] Liu S., Zhang Q., Chen C., Ge D., Qu Y., Chen R., Fan Y.-M., Li N., Tang W.W., Zhang W. (2016). Hyperinsulinemia Enhances Interleukin-17-Induced Inflammation to Promote Prostate Cancer Development in Obese Mice through Inhibiting Glycogen Synthase Kinase 3-Mediated Phosphorylation and Degradation of Interleukin-17 Receptor. Oncotarget.

[7] Bouras E., Karhunen V., Gill D., Huang J., Haycock P.C., Gunter M.J., Johansson M., Brennan P., Key T., Lewis S.J. (2022). Circulating Inflammatory Cytokines and Risk of Five Cancers: A Mendelian Randomization Analysis. BMC Med..

[8] Zhao C., Li Y., Zhang W., Zhao D., Ma L., Ma P., Yang F., Wang Y., Shu Y., Qiu W. (2018). IL-17 Induces NSCLC A549 Cell Proliferation via the Upregulation of HMGA1, Resulting in an Increased Cyclin D1 Expression. Int. J. Oncol..

[9] Li J., Panganiban R., Kho A.T., McGeachie M.J., Farnam L., Chase R.P., Weiss S.T., Lu Q., Tantisira K.G. (2020). Circulating MicroRNAs and Treatment Response in Childhood Asthma. Am. J. Respir. Crit. Care Med..

[10] Xu R., Ke X., Shang W., Liu S., Fu X., Wang T., Jin S. (2022). Distribution and Clinical Significance of IL-17A in Tumor-Infiltrating Lymphocytes of Non-Small Cell Lung Cancer Patients. Pathol. Oncol. Res..

[11] Hays L.E., Zodrow D.M., Yates J.E., Deffebach M.E., Jacoby D.B., Olson S.B., Pankow J.F., Bagby G.C. (2008). Cigarette Smoke Induces Genetic Instability in Airway Epithelial Cells by Suppressing FANCD2 Expression. Br. J. Cancer.

[12] Wu H.-H., Hwang-Verslues W.W., Lee W.-H., Huang C.-K., Wei P.-C., Chen C.-L., Shew J.-Y., Lee E.Y.-H.P., Jeng Y.-M., Tien Y.-W. (2015). Targeting IL-17B-IL-17RB Signaling with an Anti-IL-17RB Antibody Blocks Pancreatic Cancer Metastasis by Silencing Multiple Chemokines. J. Exp. Med..

[13] Kucukgul A., Isgor M.M., Duzguner V., Atabay M.N., Kucukgul A. (2020). Antioxidant Effects of Oleuropein on Hydrogen Peroxide-Induced Neuronal Stress—An In Vitro Study. Anti-Inflamm. Anti-Allergy Agents Med. Chem..

[14] Owen R.W., Giacosa A., Hull W.E., Haubner R., Würtele G., Spiegelhalder B., Bartsch H. (2000). Olive-Oil Consumption and Health: The Possible Role of Antioxidants. Lancet Oncol..

[15] Ghanbari R., Anwar F., Alkharfy K.M., Gilani A.-H., Saari N. (2012). Valuable Nutrients and Functional Bioactives in Different Parts of Olive (*Olea Europaea* L.)—A Review. Int. J. Mol. Sci..

[16] Cárdeno A., Sánchez-Hidalgo M., Rosillo M.A., Alarcón de la Lastra C. (2013). Oleuropein, a Secoiridoid Derived from Olive Tree, Inhibits the Proliferation of Human Colorectal Cancer Cell through Downregulation of HIF-1α. Nutr. Cancer.

[17] Nediani C., Ruzzolini J., Romani A., Calorini L. (2019). Oleuropein, a Bioactive Compound from *Olea Europaea* L., as a Potential Preventive and Therapeutic Agent in Non-Communicable Diseases. Antioxidants.

[18] Nardi M., Baldelli S., Ciriolo M.R., Costanzo P., Procopio A., Colica C. (2020). Oleuropein Aglycone Peracetylated (3,4-DHPEA-EA(P)) Attenuates H_2_O_2_-Mediated Cytotoxicity in C2C12 Myocytes via Inactivation of p-JNK/p-c-Jun Signaling Pathway. Molecules.

[19] Piroddi M., Albini A., Fabiani R., Giovannelli L., Luceri C., Natella F., Rosignoli P., Rossi T., Taticchi A., Servili M. (2017). Nutrigenomics of Extra-Virgin Olive Oil: A Review. BioFactors.

[20] D’Anna C., Di Sano C., Di Vincenzo S., Taverna S., Cammarata G., Scurria A., Pagliaro M., Ciriminna R., Pace E. (2022). Mesoporous Silica Particles Functionalized with Newly Extracted Fish Oil (Omeg@Silica) Reducing IL-8 Counteract Cell Migration in NSCLC Cell Lines. Pharmaceutics.

[21] Pojero F., Aiello A., Gervasi F., Caruso C., Ligotti M.E., Calabrò A., Procopio A., Candore G., Accardi G., Allegra M. (2022). Effects of Oleuropein and Hydroxytyrosol on Inflammatory Mediators: Consequences on Inflammaging. Int. J. Mol. Sci..

[22] Strøbech J.E., Giuriatti P., Erler J.T. (2022). Neutrophil Granulocytes Influence on Extracellular Matrix in Cancer Progression. Am. J. Physiol. Cell Physiol..

[23] Geyikoglu F., Isikgoz H., Onalan H., Colak S., Cerig S., Bakir M., Hosseinigouzdagani M., Koc K., Erol H.S., Saglam Y.S. (2017). Impact of High-Dose Oleuropein on Cisplatin-Induced Oxidative Stress, Genotoxicity and Pathological Changes in Rat Stomach and Lung. J. Asian Nat. Prod. Res..

[24] Kim Y.-H., Choi Y.-J., Kang M.-K., Lee E.-J., Kim D.Y., Oh H., Kang Y.-H. (2018). Oleuropein Curtails Pulmonary Inflammation and Tissue Destruction in Models of Experimental Asthma and Emphysema. J. Agric. Food Chem..

[25] Cao S., Zhu X., Du L. (2017). P38 MAP Kinase Is Involved in Oleuropein-Induced Apoptosis in A549 Cells by a Mitochondrial Apoptotic Cascade. Biomed. Pharmacother..

[26] Antognelli C., Frosini R., Santolla M.F., Peirce M.J., Talesa V.N. (2019). Oleuropein-Induced Apoptosis Is Mediated by Mitochondrial Glyoxalase 2 in NSCLC A549 Cells: A Mechanistic Inside and a Possible Novel Nonenzymatic Role for an Ancient Enzyme. Oxidative Med. Cell. Longev..

[27] Montalbano A.M., Albano G.D., Anzalone G., Moscato M., Gagliardo R., Di Sano C., Bonanno A., Ruggieri S., Cibella F., Profita M. (2020). Cytotoxic and Genotoxic Effects of the Flame Retardants (PBDE-47, PBDE-99 and PBDE-209) in Human Bronchial Epithelial Cells. Chemosphere.

[28] Albano G.D., Moscato M., Montalbano A.M., Anzalone G., Gagliardo R., Bonanno A., Giacomazza D., Barone R., Drago G., Cibella F. (2020). Can PBDEs Affect the Pathophysiologic Complex of Epithelium in Lung Diseases?. Chemosphere.

[29] Montalbano A.M., Di Sano C., Chiappara G., Riccobono L., Bonanno A., Anzalone G., Vitulo P., Pipitone L., Gjomarkaj M., Pieper M.P. (2018). Cigarette Smoke and Non-Neuronal Cholinergic System in the Airway Epithelium of COPD Patients. J. Cell. Physiol..

[30] Przychodzen P., Wyszkowska R., Gorzynik-Debicka M., Kostrzewa T., Kuban-Jankowska A., Gorska-Ponikowska M. (2019). Anticancer Potential of Oleuropein, the Polyphenol of Olive Oil, With 2-Methoxyestradiol, Separately or in Combination, in Human Osteosarcoma Cells. Anticancer. Res..

[31] Ma W., Wang Z., Zhao Y., Wang Q., Zhang Y., Lei P., Lu W., Yan S., Zhou J., Li X. (2021). Salidroside Suppresses the Proliferation and Migration of Human Lung Cancer Cells through AMPK-Dependent NLRP3 Inflammasome Regulation. Oxidative Med. Cell. Longev..

[32] Liu W., Xin M., Li Q., Sun L., Han X., Wang J. (2022). IL-17A Promotes the Migration, Invasion and the EMT Process of Lung Cancer Accompanied by NLRP3 Activation. BioMed Res. Int..

[33] Ritzmann F., Lunding L.P., Bals R., Wegmann M., Beisswenger C. (2022). IL-17 Cytokines and Chronic Lung Diseases. Cells.

[34] Møller P., Danielsen P.H., Karottki D.G., Jantzen K., Roursgaard M., Klingberg H., Jensen D.M., Christophersen D.V., Hemmingsen J.G., Cao Y. (2014). Oxidative Stress and Inflammation Generated DNA Damage by Exposure to Air Pollution Particles. Mutat. Res. Rev. Mutat. Res..

[35] Kopp B., Khoury L., Audebert M. (2019). Validation of the ΓH2AX Biomarker for Genotoxicity Assessment: A Review. Arch. Toxicol..

[36] Garcia-Canton C., Anadón A., Meredith C. (2012). ΓH2AX as a Novel Endpoint to Detect DNA Damage: Applications for the Assessment of the in Vitro Genotoxicity of Cigarette Smoke. Toxicol. Vitr. Int. J. Publ. Assoc. BIBRA.

[37] Riches L.C., Lynch A.M., Gooderham N.J. (2008). Early Events in the Mammalian Response to DNA Double-Strand Breaks. Mutagenesis.

[38] Cann K.L., Dellaire G. (2011). Heterochromatin and the DNA Damage Response: The Need to Relax. Biochem. Cell Biol..

[39] Lynch A.M., Sasaki J.C., Elespuru R., Jacobson-Kram D., Thybaud V., De Boeck M., Aardema M.J., Aubrecht J., Benz R.D., Dertinger S.D. (2011). New and Emerging Technologies for Genetic Toxicity Testing. Environ. Mol. Mutagen..

[40] Martin-Orozco N., Muranski P., Chung Y., Yang X.O., Yamazaki T., Lu S., Hwu P., Restifo N.P., Overwijk W.W., Dong C. (2009). T Helper 17 Cells Promote Cytotoxic T Cell Activation in Tumor Immunity. Immunity.

[41] Antoniou C., Hull J. (2021). The Anti-Cancer Effect of *Olea Europaea* L. Products: A Review. Curr. Nutr. Rep..

[42] ArulJothi K.N., Kumaran K., Senthil S., Nidhu A.B., Munaff N., Janitri V.B., Kirubakaran R., Singh S.K., Gupt G., Dua K. (2022). Implications of Reactive Oxygen Species in Lung Cancer and Exploiting It for Therapeutic Interventions. Med. Oncol..

[43] Dayi T., Oniz A. (2022). Effects of the Mediterranean Diet Polyphenols on Cancer Development. J. Prev. Med. Hyg..

[44] Yadegar N., Dadashi Z., Shams K., Mohammadi M., Abyar M., Rafat M. (2022). The Prominent Role of MiR-942 in Carcinogenesis of Tumors. Adv. Biomed. Res..

[45] Omar S.H. (2010). Oleuropein in Olive and Its Pharmacological Effects. Sci. Pharm..

[46] Luo Y., Liu L., Zhao J., Jiao Y., Zhang M., Xu G., Jiang Y. (2022). PI3K/AKT1 Signaling Pathway Mediates Sinomenine-Induced Hepatocellular Carcinoma Cells Apoptosis: An in Vitro and in Vivo Study. Biol. Pharm. Bull..

[47] Zheng Y., Liu Z., Yang X., Liu L., Ahn K.S. (2022). An Updated Review on the Potential Antineoplastic Actions of Oleuropein. Phytother. Res..

[48] Lee Y.S., Kim D.W., Lee Y.H., Oh J.H., Yoon S., Choi M.S., Lee S.K., Kim J.W., Lee K., Song C.-W. (2011). Silver Nanoparticles Induce Apoptosis and G2/M Arrest via PKCζ-Dependent Signaling in A549 Lung Cells. Arch. Toxicol..

[49] Scalbert A., Williamson G. (2000). Dietary Intake and Bioavailability of Polyphenols. J. Nutr..

[50] Miro-Casas E., Covas M.-I., Farre M., Fito M., Ortuño J., Weinbrenner T., Roset P., de la Torre R. (2003). Hydroxytyrosol Disposition in Humans. Clin. Chem..

[51] Bigagli E., Cinci L., Paccosi S., Parenti A., D’Ambrosio M., Luceri C. (2017). Nutritionally Relevant Concentrations of Resveratrol and Hydroxytyrosol Mitigate Oxidative Burst of Human Granulocytes and Monocytes and the Production of Pro-Inflammatory Mediators in LPS-Stimulated RAW 264.7 Macrophages. Int. Immunopharmacol..

[52] Ma R., Su H., Jiao K., Liu J. (2023). Association Between IL-17 and Chronic Obstructive Pulmonary Disease: A Systematic Review and Meta-Analysis. Int. J. Chronic Obstr. Pulm. Dis..

[53] Del Rio D., Rodriguez-Mateos A., Spencer J.P.E., Tognolini M., Borges G., Crozier A. (2013). Dietary (Poly)Phenolics in Human Health: Structures, Bioavailability, and Evidence of Protective Effects against Chronic Diseases. Antioxid. Redox Signal..

[54] Kanner J. (2023). Food Polyphenols as Preventive Medicine. Antioxidants.

